# A Differentiable Extended Kalman Filter for Object Tracking Under Sliding Regime

**DOI:** 10.3389/frobt.2021.686447

**Published:** 2021-08-09

**Authors:** Nicola A. Piga, Ugo Pattacini, Lorenzo Natale

**Affiliations:** ^1^ Humanoid Sensing and Perception, Istituto Italiano di Tecnologia, Genoa, Italy; ^2^ Dipartimento di Informatica, Bioingegneria, Robotica e Ingegneria dei Sistemi, Università di Genova, Genoa, Italy; ^3^ iCub Tech, Istituto Italiano di Tecnologia, Genoa, Italy

**Keywords:** object position tracking, object velocity tracking, differentiable extended kalman filtering, machine learning-aided filtering, humanoid robotics

## Abstract

Tactile sensing represents a valuable source of information in robotics for perception of the state of objects and their properties. Modern soft tactile sensors allow perceiving orthogonal forces and, in some cases, relative motions along the surface of the object. Detecting and measuring this kind of lateral motion is fundamental to react to possibly uncontrolled slipping and sliding of the object being manipulated. Object slip detection and prediction have been extensively studied in the robotic community leading to solutions with good accuracy and suitable for closed-loop grip stabilization. However, algorithms for object perception, such as in-hand object pose estimation and tracking algorithms, often assume no relative motion between the object and the hand and rarely consider the problem of tracking the pose of the object subjected to slipping and sliding motions. In this work, we propose a differentiable Extended Kalman filter that can be trained to track the position and the velocity of an object under translational sliding regime from tactile observations alone. Experiments with several objects, carried out on the iCub humanoid robot platform, show that the proposed approach allows achieving an average position tracking error in the order of 0.6 cm, and that the provided estimate of the object state can be used to take control decisions using tactile feedback alone. A video of the experiments is available as Supplementary Material.

## 1 Introduction

Object perception is one of the key problems of autonomous manipulation as it enables taking informed decisions based on the state of the object. Recent approaches proposed in the literature deal with the problem of estimating the 6-dimensional pose of the object from RGB (-D) images using Deep Convolutional Neural Networks (Xiang et al., 2018; Tremblay et al., 2018; Peng et al., 2019; Hodaň et al., 2020). These methods are optimal for detecting the pose of the object in absence of contacts with the end-effector but might suffer in the presence of challenging occlusion, e.g when the robot interacts with the object (Wen et al., 2020). State-of-the-art methods addressing specifically the problem of *in-hand* object tracking either use visual information and concentrates on achieving robustness to occlusions (Wen et al., 2020) or focus on providing rich and efficient tactile contact modelling (Liang et al., 2020) that can help explaining complex within-hand object motions.

Among the most typical in-hand object motions, object slipping and sliding are particularly challenging to be perceived and controlled. For this reason they have been extensively explored and studied within the literature on tactile-based perception and control. In this respect, several works (Meier et al., 2016; Veiga et al., 2018; Dong et al., 2019) propose methods, often learning-based, for slip detection and prediction and how to utilize them for grip stabilization. On the other hand, works dealing with in-hand object pose estimation and tracking do not consider the problem, as in (Wen et al., 2020). In Liang et al. (2020), a physical engine is used to model the tactile interaction with the object and integrated in an object pose tracker that explicitly takes into account slippage. The method is tested in simulation and real-world experiments. However, experimental results regarding the slippage are not provided for the real-world scenario and analyzed only in simulation.

In this work we propose an algorithm for tracking the position and the velocity of an object subjected to in-hand sliding motion using tactile observations. We do not focus on the more general problem of object slippage and we restrict our interest to pure translational sliding motions. We implemented our algorithm as a *differentiable* Kalman filter whose internal behavior is learned end-to-end from ground truth data obtained with visual feedback.

Our contributions are the following:

• We show how to model tactile sensing in the context of differentiable Kalman filtering for state tracking of an object undergoing a sliding motion while avoiding the necessity to manually write mathematically challenging motion and measurement models;
• We discuss and show experimentally the importance of differentiating over time the tactile measurements before feeding them to the learned filter;
• We provide insights on how to collect labelled data using Kalman smoothers starting from noisy ground-truth data;
• We provide position and velocity tracking performance results on experiments carried out on a real humanoid anthropomorphic hand equipped with tactile sensors.

Results show that the proposed method achieves an average position error in the order of 0.6 cm and an average velocity error in the order of 0.05 cm/s when trained on all the objects considered in our tests. We additionally provide the results of some practical experiments where the output of the learned filter is used to stop the object sliding after a given number of centimeters provided by the user.

The rest of the paper is organized as follows. After a section where we review the state of the art on both object pose tracking and slip detection and prediction using tactile sensors, we present our algorithm for object sliding tracking. We then present the results of the experiments carried out on the iCub humanoid robot platform. We conclude the paper with additional remarks on our work and possible future directions of research.

## 2 Related Work

Our work is closely related to recent advances in tactile-based object pose estimation and tracking using neural networks and deep neural networks. Given our interest in the specific problem of estimating the position of the object under sliding motion, our work is also linked to recent approaches on object slip detection and prediction.

Classical works on tactile-based object pose tracking adopted Kalman and particle filtering techniques to localize or track over time the pose of an object being manipulated by the end-effector of a robotic platform. Bimbo et al. (2015) use particles to represent the research region of an object pose estimation algorithm. The particles are initialized according to a visual prior and then particles showing high fitness with tactile data are replicated recursively. Vezzani et al. (2017) propose the Memory Unscented Particle Filter which combines an Unscented Particle Filter with a windowing based memory strategy to estimate the 6D pose of a stationary object using 3D tactile contact information. Koval et al. (2015) use tactile sensing within a Manifold Particle Filter that enforces the non-penetration constraint between the object and fingers by sampling the particles from physically plausible configurations compatible with the measured contact states.

Other works concentrated on the role of tactile sensing in recovering information on the state of an object being pushed on a plane by a manipulator. Yu and Rodriguez (2018) model the planar motion of a pushed object using the concept of limit surface that maps the forces acting on the object to its velocity under quasi-static motion regime. This motion model is then used within a real time optimization framework that fuses visual and tactile measurements. Suresh et al. (2020) extended this framework combining Gaussian process implicit surface regression and factor graph-based pose estimation in order to jointly estimate the shape and the pose of an object being pushed using tactile measurements solely.

More recent works exploit the availability of vision-based tactile sensors (Yuan et al., 2017; Lambeta et al., 2020). These sensors provide rich contact information in the form of RGB images capturing the local deformation of soft materials covering the sensor itself. Given the large availability of neural and deep neural modules for images processing, several works have combined learning techniques with the high dimensional data provided by these kind of sensors. Sodhi et al. (2020) use the Digit (Lambeta et al., 2020) vision-based tactile sensor to estimate the pose of a planar object being pushed by a manipulator. They first learn a suitable observation model that maps consecutive tactile images to the relative pose of the sensor. Then, they combine this information within a factor graph-based optimization approach for pose estimation. Very recently, Bauza et al. (2020) combined tactile images acquired with a GelSim sensor (Donlon et al., 2018) and Convolutional Neural Networks (CNN) to estimate the pose of an object in contact with the sensor from a single touch. Vision-based tactile sensors have also been adopted in (Dong et al., 2019) to detect incipient slip from changes in the motion field of the tactile images induced by the contacts with the object. This learning-free method allows detecting slip with high accuracy and has been tested in a closed-loop scenario in order to actively avoid slipping in a bottle-cap screwing/unscrewing experiment.

Although vision-based tactile sensors provide interesting performance and have been applied to a notable variety of different tasks, one problem that limit their adoption is the size of the sensors implementations. When there is the necessity to equip robotic hands with tactile sensing, other solutions based on other working principles and smaller in size have been preferred, especially in the case of anthropomorphic multi-finger hands. In this respect, Veiga et al. (2018) adopt the BioTac (Wettels et al., 2014) liquid-based deformable sensor in a supervised-learning-based algorithm that predicts the occurrence of object slipping starting from high-dimensional tactile data provided by the sensor. They also show that utilization of the predicted slip as feedback in a control algorithm allows counteracting slip events while interacting with previously unknown objects. Similarly, Sundaralingam et al. (2019) exploit estimated contact points on the surface of a BioTac sensor and the associated normals in a 3D Convolutional architecture that learns to estimate the forces exchanged between the sensor and the manipulated object. The learned force model is then adopted in a feedback grasp controller for object lifting and gentle placement.

In our work, we follow a similar path as (Veiga et al., 2018; Sundaralingam et al., 2019) as we use magnetic-based soft tactile sensors within a learning architecture in order to estimate physical quantities that explain the relative motion between the object and the sensor. However, we are not interested in detecting the slip directly or provide an interpretation of the interaction in terms of forces. Instead, we are interested in a simpler kinematic interpretation, i.e. in estimating the position and the velocity of the object while it slides between several fingers of an anthropomorphic hand, starting from the tactile measurements. To this end, we adopt a Kalman filtering approach, which makes our work related to classical works on object pose tracking and estimation. Given the complex nature of the relationship occurring between the tactile sensors and the object motion, we take inspiration from recent advances in *differentiable* Kalman filtering (Kloss et al., 2020; Lee et al., 2020) in order to learn suitable motion and measurement models from ground truth data.

## 3 Materials and Methods

### 3.1 Description of the Sensors

In this work, we study the problem of tracking the position and the velocity of an object during sliding motion from tactile measurements using differentiable, i.e. learned from data, Kalman filters. In order to better contextualize and justify the necessity to learn a tracking algorithm from data, in this section we present the sensors that we adopted in the present study.

The typology of sensors that we consider are deformable soft tactile sensors. The sensing principle is the following: when external forces deform the sensor, due to the interaction with the object, the deformation is sensed by an electronic circuit such that the change in the output signal over time is correlated with the relative motion between the sensor and the object. In the case of magnetic sensors, a magnet is embedded on the surface of the sensor, and a Hall effect sensor detects changes in the magnetic field, as pressure deforms the surface of the sensor. In particular, in this work we use the uSkin sensor (Tomo et al., 2016), and in particular a special version of the uSkin sensor that has been adapted (Holgado et al., 2019) for the anthropomorphic hands of the iCub humanoid platform (Metta et al., 2010).

**FIGURE 1:**
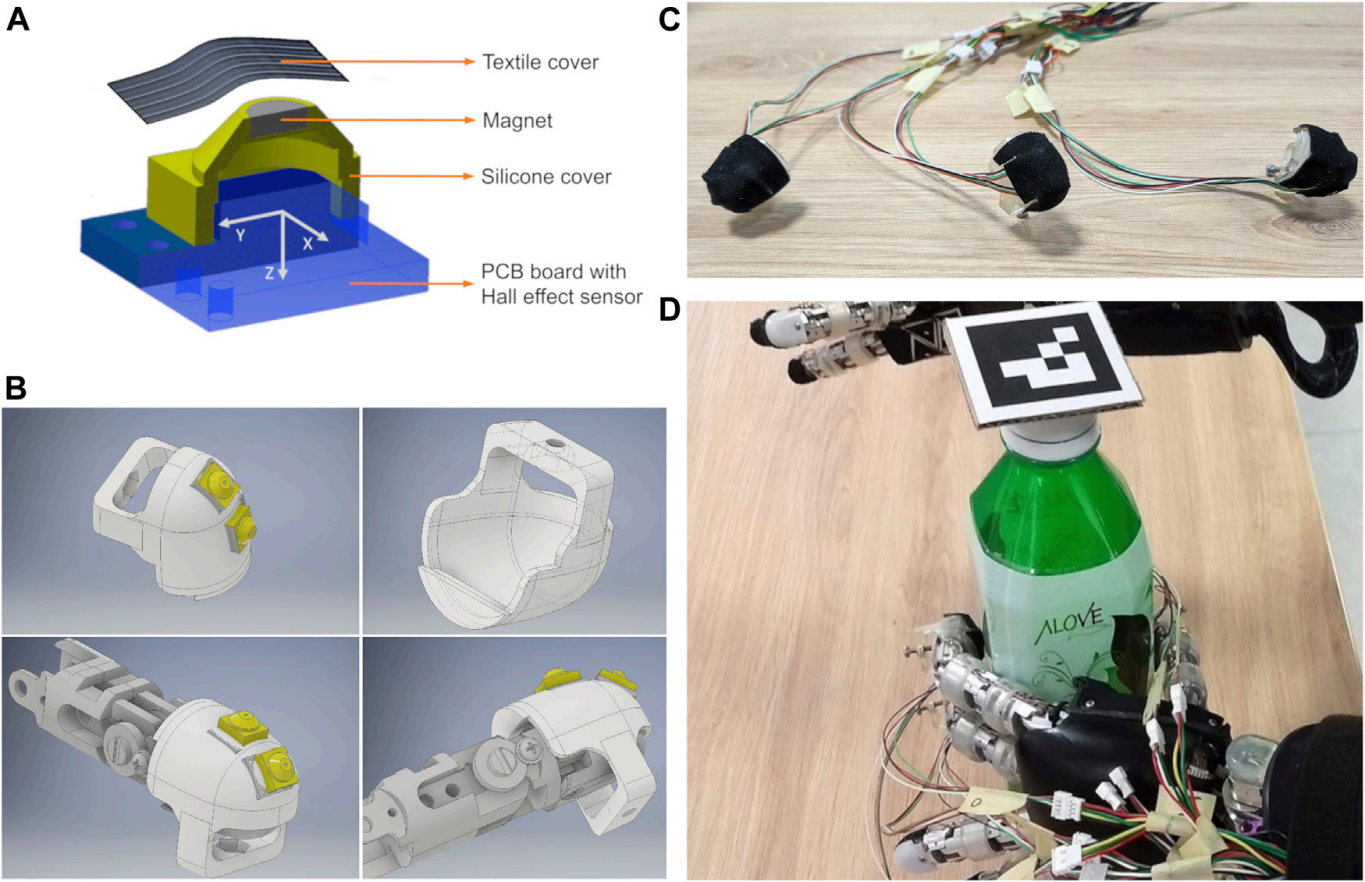
In **(A)**, the exploded view of the sensing module. In **(B)**, the CAD model of the fingertip adapter for the iCub finger hosting two sensing modules. In **(C)**, the final assembled fingertip adapters with the grip tape cover. In **(D)**, the experimental setup with the iCub humanoid robot left hand equipped with uSkin deformable soft tactile sensors.

The main sensing module of size 6 × 6 × 3.8 mm, shown in Figure 1A, consists of (from the bottom):

• a PCB board hosting the Hall effect sensor chip;
• a single soft silicone skin cover;
• a neodymium magnet;
• a flexible textile cover (realized with a high friction grip tape).

Integration of the above sensing module on the fingertips of the iCub hands is achieved with a suitable fingertip adapter (Holgado et al., 2019) (Figure 1B). The adapter hosts two sensing modules that follow the original curvature of the fingertip assembly and are mounted with an angle of 6° and 49° approximately with respect to the vector normal to the surface of the fingertip. A top layer of grip tape is finally placed over the whole assembly in order to provide good friction properties and protect the underlying sensors. The final assembly is shown in Figure 1C.

Once mounted on the fingertips of the iCub robot, the sensors allow measuring the interaction between the fingertips and the object. Specifically, when the external forces deform the silicone skin that holds the magnet, the motion of the magnet generates a change in the magnetic field that is sensed by the sensor mounted on the PCB. The actual output of the sensor consists of three channels that are proportional to the sensed magnetic field. Given the 3D nature of the output signal, the sensor output measures the following type of interactions between the object and the fingertip:

• normal interactions, such as normal forces exerted by the robot when grasping an object;
• lateral interactions, such as lateral and shear forces occurring when the object slides between the fingers of the robot.

In the present study, we are interested in exploiting the 3D nature of the tactile signals in order to track over time the position and the velocity of an object while it is involved in a sliding motion between the fingertips of the multi-fingered anthropomorphic hand. Given the considerable complexity of the relationships occurring between the object motion and the output of the sensors, involving, among the others, the physics of rigid and elastic objects and that of magnetic fields, we propose to adopt a Machine Learning signal filtering approach to tackle this problem.

### 3.2 Problem Definition

Given a stream of noisy tactile measurements ${\left\{\tau_{t}\right\}}_{1\leq t\leq N_{\tau }}$, our goal is to track the 1D position of the object *p*_*t*_ and its velocity *v*_*t*_ along the *z* coordinate of the robot root frame while the object is sliding between the fingertips of the robot hand. We are not interested in solving the localization problem for the object, hence we assume that *p*_0_ = 0. A complete description of the state of the object is given by the state vector

$$x_{t}=\left(\begin{array}{c} p_{t}\\ v_{t} \end{array}\right)\in R^{n},$$ (1)

where *n* = 2 is the state size. In order to track the state *x*_*t*_, we adopt a Kalman filtering approach (Kalman, 1960) that approximates the posterior distribution of the state given the tactile measurements up to time *t p* (*x*_*t*_|*τ*_1:*t*_) with a Gaussian distribution

$$\hat{p}\left(x_{t}|\tau_{1:t}\right)=N\left(x_{t};\mu_{t},P_{t}\right).$$ (2)

The belief on the state of the object is updated recursively by means of a prediction step, that leverages prior information in the form of a predefined *motion model*, and a correction step, that incorporates a new tactile measurement *τ*_*t*_ according to a predefined *measurement model*.

Motion and measurement models are usually *first principles models*, i.e. they are obtained form the combination of suitable physical laws that describe the system of interest. However, deriving such models is not always possible if the system is considerably complex or the working principles are not completely known. This is the case for the majority of the tactile sensors adopted in the literature. Nevertheless, recent advances in differential Kalman filtering (Haarnoja et al., 2016; Kloss et al., 2020), allows *learning* the required models from experimental data.

In this work, we decided to adopt a differentiable Extended Kalman Filtering framework (dEKF), for the following reasons:

• the possibility to *learn* motion and measurement models for the adopted tactile sensing system from ground truth data in the form of state-dependant neural networks;
• the ability of the EKF to handle non-linear relationships between state and measurements, as those modeled by neural networks;
• the recognized superiority (Kloss et al., 2020; Lee et al., 2020) in terms of performance and interpretability of differential Kalman filters over unstructured alternatives like LSTMs.

In the remainder of this section, we describe the proposed algorithm in details.

### 3.3 Extended Kalman Filtering

In this section we recall the working principles of the Extended Kalman Filter algorithm for tracking of the state vector *x*_*t*_ given generic measurements *z*_*t*_. The algorithm assumes that the belief about the state is modelled as the posterior distribution *p* (*x*_*t*_|*z*_1:*t*_), given all the measurements *z*_1:*t*_ up to the instant of time *t*. The posterior is approximated using a Gaussian distribution

$$\hat{p}\left(x_{t}|z_{1:t}\right)=N\left(x_{t};\mu_{t},P_{t}\right),$$ (3)

under the assumption that the state *x*_*t*_ evolves according to a Markovian dynamic model of the form

$$p\left(x|x_{t-1}\right)=N\left(x_{t};f\left(x_{t-1}\right),Q_{t}\right),$$ (4)

and the measurements *z*_*t*_ are conditionally independent given *x*_*t*_ and normally distributed, i.e.

$$p\left(z_{t}|x_{1:t},z_{1:t-1}\right)=p\left(z_{t}|x_{t}\right)=N\left(z_{t};h\left(x_{t}\right),R_{t}\right).$$ (5)

*f* and *h* are generic non-linear differentiable functions while *Q*_*t*_ and *R*_*t*_ are referred as the process and measurement noise covariance matrix respectively. Eq. 4 and Eq. 5 can be expressed in functional form, resulting in the following motion model

$$\begin{array}{cc} x_{t} & =f\left(x_{t-1}\right)+w_{t-1},\\ w_{t} & ∼N\left(0,Q_{t}\right), \end{array}$$ (6)

and measurement model

$$\begin{array}{cc} z_{t} & =h\left(x_{t}\right)+\nu_{t},\\ \nu_{t} & ∼N\left(0,R_{t}\right). \end{array}$$ (7)

At each instant of time *t*, the previous belief

$$N\left(x;\mu_{t-1},P_{t-1}\right)$$ (8)

is updated according to the model in Eq. 6 producing the predicted mean $\mu_{t}^{-}$ and covariance $P_{t}^{-}$ of the state:

$$\begin{array}{cc} \mu_{t}^{-} & =f\left(\mu_{t-1}\right)\\ P_{t}^{-} & =F_{x}\left(\mu_{t-1}\right)P_{t-1}F_{x}{\left(\mu_{t-1}\right)}^{T}+Q_{t-1} \end{array}$$ (9)

where $F_{x}\left(\mu \right)=\frac{\partial f\left(x\right)}{\partial x}|{\left.\right|}_{x=\mu }$. A new measurement *z*_*t*_ is then incorporated via the measurement model in Eq. 7 according to the following correction step:

$$\begin{array}{cc} P_{z,t} & =H_{x}\left(\mu_{t}^{-}\right)P_{t}^{-}H_{x}{\left(\mu_{t}^{-}\right)}^{T}+R_{t},\\ K_{t} & =P_{t}^{-}H_{x}{\left(\mu_{t}^{-}\right)}^{T}{\left(P_{z,t}\right)}^{-1},\\ z_{t}^{-} & =h\left(\mu_{t}^{-}\right),\\ \mu_{t} & =\mu_{t}^{-}+K_{t}\left(z_{t}-z_{t}^{-}\right),\\ P_{t} & =P_{t}^{-}-K_{t}P_{z,t}K_{t}^{T}. \end{array}$$ (10)

*K*_*t*_ is usually called the Kalman gain, *P*_*z*,*t*_ is the measurement covariance matrix, $z_{t}^{-}$ is the predicted mean of the measurement and $H_{x}\left(\mu \right)=\frac{\partial h\left(x\right)}{\partial x}|{\left.\right|}_{x=\mu }$. The actual estimate ${\hat{x}}_{t}$ is extracted as the mean *μ*_*t*_ of the approximate posterior $\hat{p}\left(x|z_{1:t}\right)$.

### 3.4 Object Motion Modelling

The motion model in Eq. 6 provides a priori information on the state of the object at time *t* given the state at the previous instant of time *t*−1. We adopted a hybrid approach that combines the physical notions of position and velocity with a neural network. Our models is as follows:

$$\begin{array}{cc} x_{t} & =\left(\begin{array}{c} p_{t}\\ v_{t} \end{array}\right)=f\left(x_{t-1}\right)+w,\\ & =\left(\begin{array}{c} p_{t-1}+v_{t-1}\Delta_{T}\\ v_{t-1}+NN_{\theta_{1}}\left(x_{t-1}\right) \end{array}\right)+w,\\ w & ∼N\left(0,Q\right). \end{array}$$ (11)

The positional part *p*_*t*_ is updated using a constant velocity model (Bar-Shalom et al., 2002) where the elapsed time Δ_*T*_ is assumed equal to the sampling period of the algorithm. Instead, the change in velocity between consecutive instant of times *v*_*t*_ − *v*_*t*−1_ is modelled as the output of a neural network $NN_{\theta_{1}}$ with parameters *θ*_1_ given the state at the previous instant of time *x*_*t*−1_:

$$v_{t}-v_{t-1}=NN_{\theta_{1}}\left(x_{t-1}\right).$$ (12)

The process noise covariance matrix $Q\in R^{2\times 2}$ is also considered as a parameter to be trained.

The rationale behind our choice is the following. For the positional part, we know that the position is the integral of the velocity. Therefore we can utilize this knowledge in the model without the necessity to learn it from the data. Conversely, the change in velocity during a sliding motion might be challenging to model from first principles, and for this reason, it is preferable to model it with a neural network that is trained using ground-truth data.

The description of the motion model is completed by the specification of the Jacobian *F*_*x*_(*μ*) that is needed for the execution of the Kalman prediction and correction steps. It can be readily evaluated as:

$$F_{x}\left(\mu \right)=\left[\begin{array}{cc} 1 & \Delta_{T}\\ \frac{\partial NN_{\theta_{1}}\left(p,v\right)}{\partial p}|{\left.\right|}_{\left(p,v\right)=\mu } & 1+\frac{\partial NN_{\theta_{1}}\left(p,v\right)}{\partial v}|{\left.\right|}_{\left(p,v\right)=\mu } \end{array}\right].$$ (13)

Here, *p*(*μ*) and *v*(*μ*) are the positional and velocity part of the mean *μ* respectively. The partial derivatives $\frac{\partial NN_{\theta_{1}}\left(p,v\right)}{\partial p}|{\left.\right|}_{\left(p,v\right)=\mu }$ and $\frac{\partial NN_{\theta_{1}}\left(p,v\right)}{\partial v}|{\left.\right|}_{\left(p,v\right)=\mu }$ can be evaluated using automatic differentiation once the structure of the neural network $NN_{\theta_{1}}$ is defined.

### 3.5 Tactile Measurements Modelling

The measurement model in Eq. 7 provides a description of the relationship between the state of interest and the output of the sensors. In this work, the adopted sensors are tactile sensors, described in Section 3.1, each producing a 3D signal

$$\tau_{j}=\left(\begin{array}{c} \tau_{j,x}\\ \tau_{j,y}\\ \tau_{j,z} \end{array}\right)\in R^{3},$$ (14)

proportional to the displacement of the *j*-th sensor elastic membrane during the interaction with the object. Given *L* tactile sensors, they can be expressed in a single vector

$$\tau =\left(\begin{array}{c} \tau_{1}\\ \vdots \\ \tau_{N} \end{array}\right)\in R^{3L}.$$ (15)

We remark that out approach does not depend on the number of signals produced by the single tactile sensor, hence in the following we will refer to $\tau \in R^{ML}$ as the vector of the signals produced by all the sensors where *M*, in the specific case of the adopted sensors, resolves to 3. Conversely, we make the assumption that the output of the sensors does not change over time if there is no relative motion between the object and the sensors. In this respect, we claim that it would be difficult to associate the tactile measurements directly to the object state, especially the velocity part, using an instantaneous relationship as in Eq. 7. Indeed, if the object does not move, the signal of the sensor is constant, and any possible value assumed by the sensor would need to be associated with a zero velocity. Given that this kind of association is ill-posed we propose instead to use, as a measurement, the first derivative $\dot{\tau }$ of the tactile signal. As a consequence, if the signal of the sensor does not change it can be unambiguously associated with a zero velocity and vice versa.

In order to specify the actual measurement function *h*(*x*), considering the complexity of the measurement process that depends on the specific nature of the tactile sensors and their working principle, we propose to use a neural network $NN_{\theta_{2}}$ with parameters *θ*_2_ to be trained using ground truth data. The resulting measurement model is:

$$\begin{array}{cc} z_{t} & =NN_{\theta_{2}}\left(x_{t}\right)+\nu \in R,\\ \nu & ∼N\left(0,R\right). \end{array}$$ (16)

The measurement noise covariance matrix R∈R is also considered as a parameter to be trained.

The goal of the network $NN_{\theta_{2}}$ is to predict a 1-dimensional feature given the state *x*. In order to make the feature comparable with the actual measurement $\dot{\tau }$, we employ a secondary network $NN_{\theta_{3}}$ with parameters *θ*_3_ that maps the measurement $\dot{\tau }$ to a measured feature $z=NN_{\theta_{3}}\left(\dot{\tau }\right)$. Given the above assumptions, the Kalman correction step in Eq. 10 becomes

$$\begin{array}{cc} P_{z,t} & =H_{x}\left(\mu_{t}^{-}\right)P_{t}^{-}H_{x}{\left(\mu_{t}^{-}\right)}^{T}+R,\\ K_{t} & =P_{t}^{-}H_{x}{\left(\mu_{t}^{-}\right)}^{T}{\left(P_{z,t}\right)}^{-1},\\ z_{t} & =NN_{\theta_{3}}\left({\dot{\tau }}_{t}\right),\\ z_{t}^{-} & =NN_{\theta_{2}}\left(\mu_{t}^{-}\right),\\ \mu_{t} & =\mu_{t}^{-}+K_{t}\left(z_{t}-z_{t}^{-}\right),\\ P_{t} & =P_{t}^{-}-K_{t}P_{z,t}K_{t}^{T}. \end{array}$$ (17)

The description of the measurement model is completed with the specification of the Jacobian $H_{x}\left(\mu \right)=\frac{\partial NN_{\theta_{2}}\left(x\right)}{\partial x}|{\left.\right|}_{x=\mu }$ that can be obtained analytically using automatic differentiation.

In summary, the adopted measurement model uses two neural networks in order to 1) map the actual measurement $\dot{\tau }$ to a 1-dimensional measured feature *z* and 2) map the predicted state $\mu_{t}^{-}$ to a 1-dimensional predicted feature *z* ^−^. The two networks are not trained separately as the target feature and its characteristics are not known a priori. Instead, they are trained jointly such that the best intermediate feature for the filtering task is learned from ground truth data.

### 3.6 Tracking Framework

The tracking process can start after the specification of suitable initial conditions for the mean of the state *μ*_0_ and the associated covariance *P*_0_. We are not interested in localizing the object, i.e. knowing its absolute position in the robot root frame, rather to its relative motion from the beginning of the experiment. For this reason, we initialize the positional part of the mean *p* (*μ*_0_) to zero. Regarding the velocity part, we do not assume any external source of information that can provide insights on the initial object velocity. As a consequence, we also set the initial velocity *v* (*μ*_0_) to zero.

**FIGURE 2:**
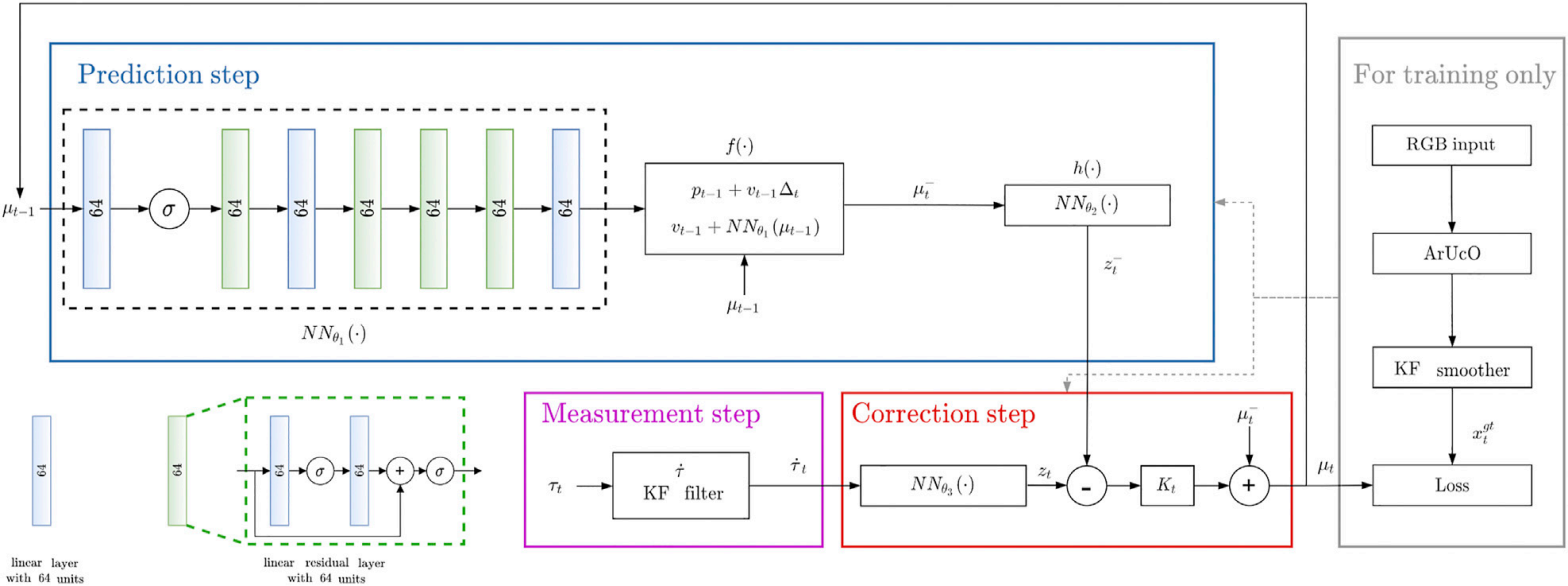
Overview of the proposed differentiable filtering architecture for object sliding tracking. *σ* indicates the ReLU activation function.

An overview of the proposed architecture is presented in Figure 2.

### 3.7 Training Procedure

The set of parameters of the differentiable filter that need to be trained are:

• The weights *θ*_1_ of the neural network that models the velocity increments of the object as part of the motion model in Eq. 11;
• The weights *θ*_2_ of the neural network that extracts feasible measurement predictions, given the predicted state, as part of the measurement model in Eq. 16;
• The weights *θ*_3_ of the neural network that extracts feasible measurement features from the actual tactile measurement $\dot{\tau }$;
• The process noise covariance matrix Q associated to the motion model in Eq. 11;
• The measurement noise covariance matrix R associated to the measurement model in Eq. 16.

In order to train the parameters *θ*_1_, *θ*_2_, *θ*_3_, *Q* and *R* we adopted an end-to-end learning procedure. Following prior work (Lee et al., 2020), we assume the following inputs to the procedure:

• a set *X* of *N* ground truth sequences $\left\{x_{t,i}^{gt}\right\}$, where *i* is the sequence index, containing the full state *x*_*t*_ of the object involved in a sliding motion. Each trajectory is *T* steps long;
• a set *Y* of *N* measurement sequences $\left\{{\dot{\tau }}_{t,i}\right\}$ that are compatible with the states *x*_*t*,*i*_ both physically and in terms of the signal length *T*.

Each sequence in *X* and *Y* is then divided in sub-sequences of length *T*_*s*_ < *T*. Each sub-sequence starts at *t* = *t*_0,*s*_ with *s* indicating a sub-sequence. Given a batch size *B* < *N* and a selection *X*_*B*_ of *B* sequences among the *N* available, a set of *B* filters, that share the same parameters, are initialized with samples extracted from the ground truth distributions

$$p\left(x_{t,i}^{gt}\right)∼N\left(x_{t,i}^{gt},P_{0}\right),$$ (18)

with *t* = *t*_0,*s*_ and *i* ∈ *X*_*B*_. Then, the state of the filters is updated using the associated measurements from *Y*_*B*_ for *T*_*s*_ steps *via* Kalman prediction and correction. The performance on a given sub-sequence is evaluated using a MSE loss of the form:

$$L_{s}=\frac{1}{\left(T_{S}-1\right)\times B\times n}\underset{i\in X\left(B\right)}{\sum }\left(\overset{t_{0,s}+T_{s}}{\underset{t=t_{0,s}+1}{\sum }}{\left(x_{t,i}^{gt}-\mu_{t,i}\right)}^{T}\left(x_{t,i}^{gt}-\mu_{t,i}\right)\right).$$ (19)

The loss *L*_*s*_ is finally used to correct the parameters *θ*_1_, *θ*_2_, *θ*_3_, *Q* and *R* via back-propagation. When the procedure has been repeated for all the sub-sequences and for all the possible choices of *B* sequences among the *N* available, the training epoch is completed and a new epoch can be processed.

## 4 Implementation Details

In this section we provide essential details necessary to train the proposed differential filter for object position and velocity tracking. After a short description of the iCub humanoid platform in Section 4.1, we describe in Section 4.2 how we collected the training and testing data via experiments of controlled sliding of several objects. Next, in Section 4.3 we explain how the collected data have been post-processed in order to be used as ground-truth data for the training and testing procedure. Finally, in Section 4.4, we describe the structure of the networks $NN_{\theta_{1}}$, $NN_{\theta_{2}}$ and $NN_{\theta_{3}}$ that represent a core part of the proposed differentiable filter and the adopted training strategy.

### 4.1 iCub Humanoid Platform

The robot platform adopted in this work is the iCub humanoid platform (Metta et al., 2010). The hands of the iCub are endowed with nine joints that can be controlled using several control modes in order to decide the position of the fingertips. In this work, we only control the position of the thumb, index and middle fingers using voltage inputs. Each finger is equipped with two uSkin sensing modules mounted on the adapter that we described in Section 3.1. Furthermore, a RGB camera system is mounted on the head of the robot in order to track with ArUco markers (Garrido-Jurado et al., 2014) the actual position of the object so to collect training data and evaluate the performance of the algorithm. The experimental setup is shown in Figure 1D. In the top left panel of the same figure, we report the reference frame attached to each sensing module.

### 4.2 Dataset Collection Procedure

In order to train the networks $NN_{\theta_{1}}$ in Eq. 11, $NN_{\theta_{2}}$ and $NN_{\theta_{3}}$ in Eq. 17 and the trainable covariance matrices *Q* in Equation 11 and *R* in Eq. 16, it is required to collect a set *X* of ground truth sequences $\left\{x_{t,i}^{gt}\right\}$ of the object of interest under sliding motion and the associated set *Y* of tactile measurements $\left\{{\dot{\tau }}_{t,i}\right\}$. To this end, we setup a repeatable controlled sliding experiment using the thumb, index and middle fingers of the iCub robot hand. In this experiment, the object of interest is manually placed on a table in front of the robot in a fixed starting pose. The arm of the robot is moved near the object, in a fixed pose, such that the hand can grasp the object. Once the object has been grasped, the robot arm moves up and then the fingers are controlled in a way such that the object either remains approximately stationary or slides between the fingers.

#### 4.2.1 Controlled Object Sliding

We are not interested in precisely control the object sliding, i.e. its position or its velocity during the sliding, rather to collect data on any kind of object sliding in terms of object position and velocity, along the direction orthogonal to the table, and sensors output. For this reason, we developed a simple PI closed-loop control algorithm in order to indirectly control the force exerted by each finger on the surface of the object held by the robot hand. Considering the kinematics of the iCub robot hand, the placements of the two sensors on each finger and the reference frame attached to each sensor (as in Figure 1A), we found experimentally that the object sliding could be controlled by changing the grip force, indirectly affected by regulating the *z* channel of the first sensor mounted on the index and middle fingers, in the following indicated as $\tau_{z}^{index}$ and $\tau_{z}^{middle}$, and the *z* channel of the second sensor mounted on the thumb finger, in the following $\tau_{z}^{thumb}$. In fact, these channels are proportional to the displacement of the sensor membrane in a direction that is approximately orthogonal to the surface of the object. Hence, regulation of those signals allow deciding the degree of stability of the grasp and, eventually, to start and stop the object sliding.

In order to regulate each signal to a desired set-point, we designed three independent closed-loop control systems, one per finger, using a proportional-integral (PI) controller running at 100 Hz. Specifically, we set the voltage *V*_*f*_ of the proximal joint of the finger according to the following control law:

$$V_{f}=k_{p,f}\left(\tau_{z,des}^{f}-\tau_{z}^{f}\right)+k_{i,f}\int \left(\tau_{z,des}^{f}-\tau_{z}^{f}\right)\;dt,$$ (20)

where *f* ∈ {index, middle, thumb}, *k*_*p*,*f*_ is the proportional gain for the finger *f*, *k*_*i*,*f*_ is the integral gain for the finger *f*, $\tau_{z}^{f}$ is the *z* tactile channel for the finger *f* and $\tau_{z,des}^{f}$ is the desired value for the finger *f*. The distal joints of each finger, instead, have been regulated to suitable configurations using the iCub built-in joint position control loops.

We remark that, in order to execute the controlled sliding experiment, we adopted specific precautions to be sure that when releasing the object, after modifying the grasp strength, it would only slide downwards without unregulated rotations. Specifically, we added weight to each object in order move the center of mass of the object below the contact points and we chosen the position of the object such that contact points, resulting from the grasp, would not allow the development of unbalanced torques.

#### 4.2.2 Design of Grasp Strength Trajectories

The choice of the desired values $\tau_{z,des}^{f}$ has been realized taking inspiration from a former work on hierarchical grasp control using tactile feedback (Regoli et al., 2016). Given a desired grasp strength *g*_*des*_ in Newton, in this work the forces $F_{des}^{f}$ exerted by the fingers on the object are partitioned as follows:

$$F_{des}^{index}=F_{des}^{middle}=\frac{g_{des}}{2},\;F_{des}^{thumb}=g_{des}.$$ (21)

We replicated a similar reasoning by defining a generalized grasp strength in the space of the sensors output, *τ*_*g*,*des*_, and assigning the following desired values:

$$\tau_{z,des}^{index}=\tau_{z,des}^{middle}=\frac{\tau_{g,des}}{2},\;\tau_{z,des}^{thumb}=\tau_{g,des}.$$ (22)

**FIGURE 3:**
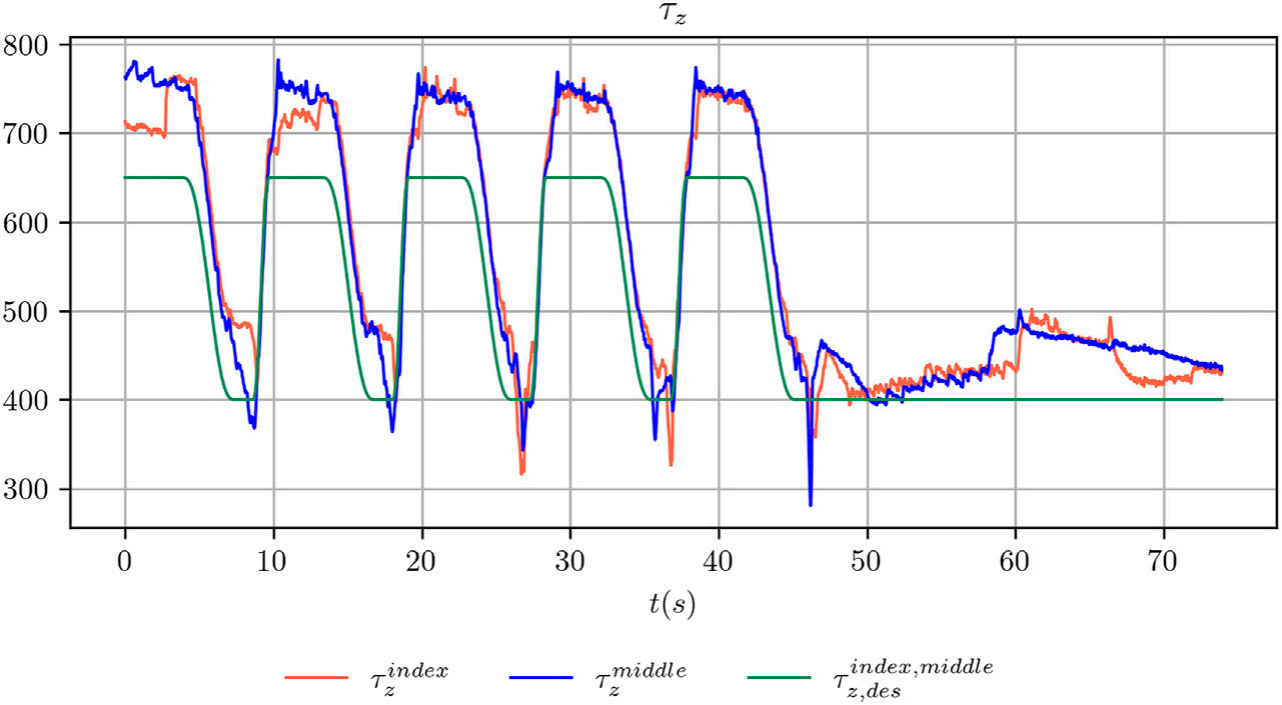
Comparison between the desired grasp strength and the achieved grasp strength for the index and middle fingers during the execution of a controlled sliding experiment.

**FIGURE 4:**
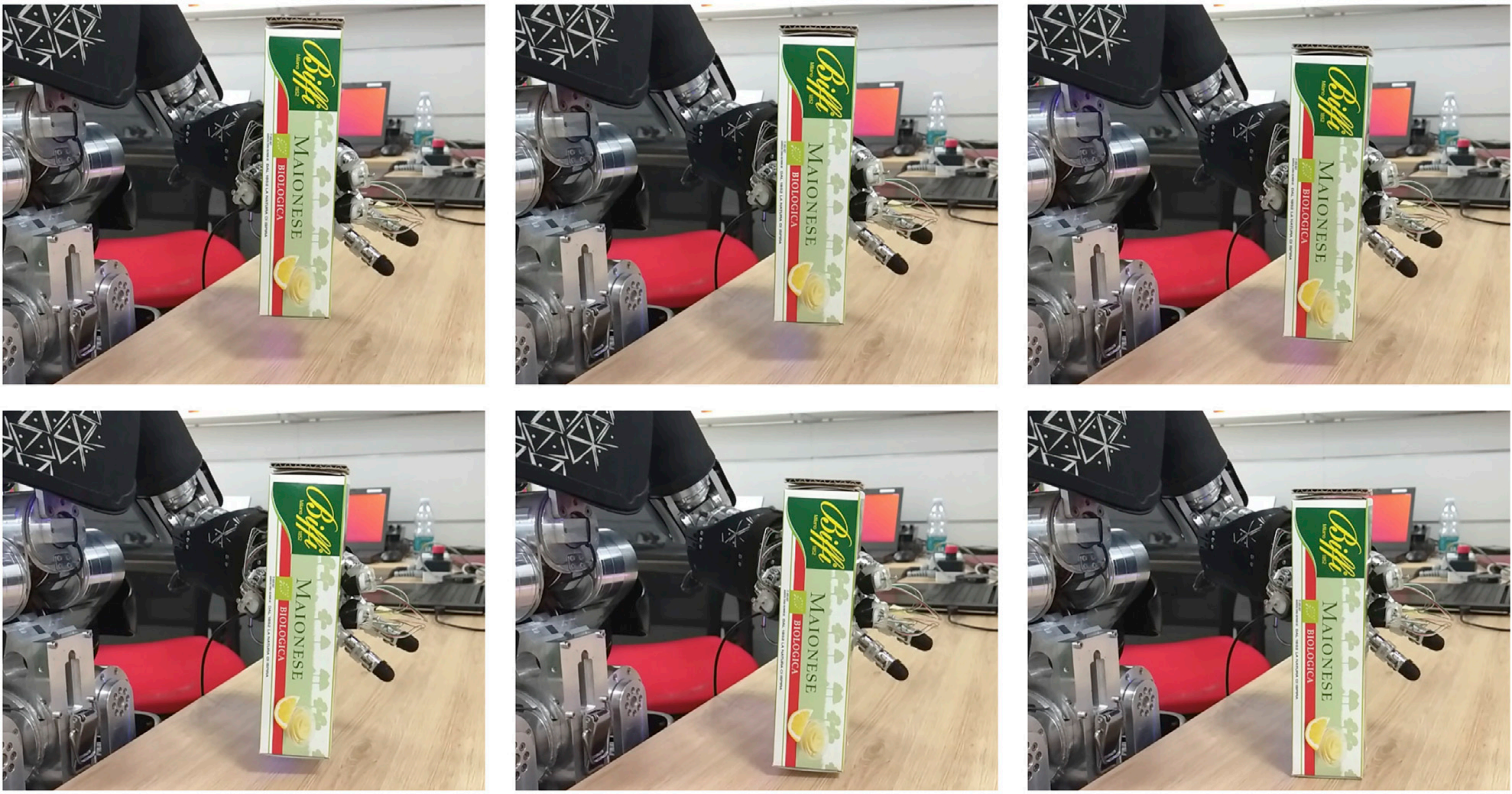
Outcome of the controlled sliding experiment with the box-shaped object.

We concluded the design of the desired references by assigning a periodic smooth trajectory to the desired grasp strength *τ*_*g*,*des*_ such that it moves alternatively between a maximum value *τ*_*g*,*max*_ and a minimum value *τ*_*g*,*min*_. The two values have been chosen, for each object considered in the present study, such that when the grasp strength is regulated to the maximum value the object does not move. Conversely, when it is regulated to the minimum value the object slides noticeably between the fingers of the robot hand. An example of the commanded and achieved trajectory for the index and middle fingers is shown in Figure 3. Figure 4 shows the outcome of the controlled sliding experiment using a box-shaped object.

#### 4.2.3 Description of the Collected Data

During each experiment, the position of the object $p_{t}^{A}\in R$, expressed in the robot root frame and projected along the direction orthogonal to the table, is acquired at 30 Hz using an ArUco marker placed on the top of the object such that it is visible from the RGB camera system mounted on the head of the robot. All the tactile signals coming from the setup are also recorded at the same frequency. We collected the *x*, *y* and *z* coordinates of the first sensor mounted on the index and middle fingers and the same coordinates of the second sensor mounted on the thumb finger in a vector $\tau \in R^{9}$ composed as follows:

$$\tau =\left(\begin{array}{c} \tau^{index}\\ \tau^{middle}\\ \tau^{thumb} \end{array}\right)$$ (23)

where $\tau^{index}\in R^{3}$, $\tau^{middle}\in R^{3}$ and $\tau^{thumb}\in R^{3}$. Our choice to use the first sensor for the index and middle fingers and the second sensor for the thumb finger depends on the fact that, given the kinematic structure of the iCub hand and specific experiment that we designed, these are the specific sensors that are actually in contact with the surface of the object.

### 4.3 Data Post-Processing

Before the collected data can be used for training purposes, it needs to be further processed as explained in the following.

#### 4.3.1 Object Position and Velocity

As explained in Section 3.7, the training procedure requires the availability of the ground truth state of the object $x_{t,i}^{gt}$ for each experiment *i*. Although the position of the object $p_{t,i}^{A}$, acquired using an ArUco marker, is available, it cannot be directly employed. In fact, the signal produced by the marker estimation system is considerably noisy to be used as a label. Furthermore, it does not provide the velocity of the object that we require.

In order to provide an almost noise-free ground-truth signal for both the position and the velocity of the object, we relied on a linear Kalman Smoother (Bar-Shalom et al., 2002). This algorithm, given suitable motion and measurement models, provide an estimate $x_{t}^{sm}$ of the state of interest at each instant of time *t* given all the measurements *z*_0:*T*_ up to the final instant of time *T*. Using all the measurements is possible because the smoothing procedure is executed offline after the actual data collection. In our case, we set the state to be smoothed $x_{t}^{sm}$ to be equal to the state of interest *x*_*t*_ as defined in Eq. 1:

$$x_{t}^{sm}=\left(\begin{array}{c} p_{t}^{sm}\\ v_{t}^{sm} \end{array}\right)\in R^{2}.$$ (24)

As motion model, we adopted a simple constant velocity model with Gaussian noise (Bar-Shalom et al., 2002):

$$\begin{array}{cc} x_{t}^{sm} & =\left(\begin{array}{c} p_{t}^{sm}\\ v_{t}^{sm} \end{array}\right)=\left(\begin{array}{c} p_{t-1}^{sm}+v_{t-1}^{sm}\Delta_{T}\\ v_{t-1}^{sm} \end{array}\right)+w^{sm},\\ w^{sm} & ∼N\left(0,Q^{sm}\right) \end{array}$$ (25)

with Δ_*T*_ the sampling time of the algorithm.

We drive the Kalman smoother using the ArUco estimate $p_{t}^{A}$ as the measurement $z_{t}^{sm}$ (after removing the first sample from the signal such that the position of the object starts from zero). The measurement process is easily modelled as:

$$\begin{array}{cc} z_{t}^{sm} & =p_{t}^{sm}+\nu^{sm},\\ \nu^{sm} & ∼N\left(0,R^{sm}\right). \end{array}$$ (26)

Given the smoothed state $x_{t,i}^{sm}$ for the *i*-th experiment, we define the ground-truth trajectory $x_{t,i}^{gt}$ as

$$x_{t,i}^{gt}≔x_{t,i}^{sm}.$$ (27)

**FIGURE 5:**
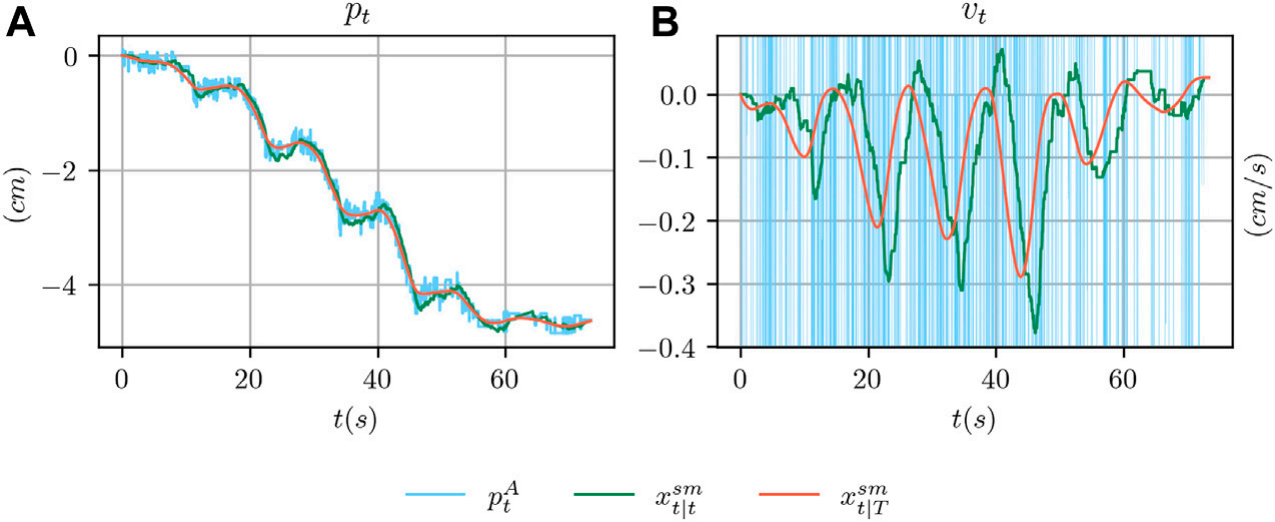
In **(A)**, comparison between the ground truth position from the ArUco marker detection system $p_{t}^{A}$, its filtered version $p_{t|t}^{sm}$ and its smoothed version $p_{t|T}^{sm}$ obtained using a Kalman filter and smoother respectively. In **(B)**, comparison between the ArUco velocity signal obtained using finite differences, filtered and smoothed version.

An example of the outcome of the smoothing procedure is shown in Figure 5 where the ArUco estimate is compared with the smoothed estimate $x_{t|T}^{sm}$ and the estimate $x_{t|t}^{sm}$ that we could have obtained using a linear Kalman filter. For the velocity component, we also compare with the velocity obtained by finite differentiation of $p_{t}^{A}$. As it can be seen, the smoothed estimate contain less noise than the ArUco estimate and the filtered estimate. Furthermore, the smoothed velocity $v_{t|T}^{sm}$ has less delay than the filtered counterpart and is significantly more reliable than using finite differences.

#### 4.3.2 Derivative of Tactile Measurements

As discussed in Section 3.5, using the tactile signal *τ*_*t*_ as a measurement would result in an ill-posed measurement function *z*_*t*_ = *h* (*τ*_*t*_). For this reason, the first derivative of the tactile signal ${\dot{\tau }}_{t}$ is used instead. Given that the actual sensors do not provide the derivative as one of the outputs, we decided to adopt a linear Kalman filter to estimate the derivative. The state of interest is given by

$$x_{t,\tau }=\left(\begin{array}{c} {\hat{\tau }}_{t}\\ {\hat{\dot{\tau }}}_{t} \end{array}\right)\in R^{18}$$ (28)

We adopted a simple constant velocity motion model of the form:

$$\begin{array}{cc} x_{t,\tau } & =\left(\begin{array}{c} {\hat{\tau }}_{t}\\ {\hat{\dot{\tau }}}_{t} \end{array}\right)=\left(\begin{array}{c} {\hat{\tau }}_{t-1}+{\hat{\dot{\tau }}}_{t-1}\Delta_{T}\\ {\hat{\dot{\tau }}}_{t-1} \end{array}\right)+w_{\tau },\\ w_{\tau } & ∼N\left(0,Q_{\tau }\right) \end{array}$$ (29)

with Δ_*T*_ the sampling time of the algorithm. The filter is fed with the tactile measurements *z*_*t*,*τ*_ = *τ*_*t*_ that are easily modelled with the following measurement process:

$$\begin{array}{cc} z_{t,\tau } & ={\hat{\tau }}_{t}+\nu_{\tau },\\ \nu_{\tau } & ∼N\left(0,R_{\tau }\right). \end{array}$$ (30)

The filtered derivative ${\hat{\dot{\tau }}}_{t}$ is evaluated for each experiment *i* so as to provide the set *Y* of the measurement sequences $\left\{{\dot{\tau }}_{t,i}\right\}$ required for the training process as per Section 3.7, i.e.

$${\dot{\tau }}_{t,i}≔{\hat{\dot{\tau }}}_{t,i}.$$ (31)

We remark that the filtered derivative is also used online, once the differentiable filter has been trained, to provide the input measurements to the algorithm.

#### 4.3.3 Data Normalization

We perform data normalization on each scalar component of the tactile measurement ${\dot{\tau }}_{t,i}\in R^{9}$ separately by dividing it by the maximum absolute value of the component among all frames *t* ∈ {0, … , *T*} and all experiments *i* ∈ {0, … , *N*} such that the normalized components are within −1 and +1.

We applied a similar procedure on the ground truth state $x_{t,i}^{gt}$. Specifically, we divided the velocity component $v_{t,i}^{gt}$ by the maximum velocity among all frames and all experiments such that the normalized velocity is between −1 and +1. Given that the position and the velocity are connected by a differential relationship, i.e

$$v_{t,i}^{gt}=\frac{d}{dt}x_{t,i}^{gt},$$ (32)

we divided the position component $p_{t,i}^{gt}$ by the same maximum velocity, to avoid altering such relationship.

### 4.4 Network Architectures

In this section we provide a concise description of the inner architecture of the networks $NN_{\theta_{1}}$, $NN_{\theta_{2}}$ and $NN_{\theta_{3}}$. Furthermore, we explain how we handle the trainable noise covariance matrices *Q* and *R*.

#### 4.4.1 Neural Networks

We followed prior work of Lee et al. (2020) to take inspiration for the design of the architectures. For the network $NN_{\theta_{1}}$ in Eq. 11, we first feed the state *x*_*t*−1_ through a multi-layer encoder with the following structure:

• 1 linear layer with 64 units;
• 1 ReLU activation;
• 1 linear residual layer.
The extracted feature is then passed to a shared stage composed by:
• 1 linear layer with 64 units;
• 3 linear residual layers;
• 1 linear layer with 64 units with output size equal to 1,
where the structure of the linear residual layer is as follows:
• 1 linear layer with 64 units;
• 1 ReLU activation;
• 1 linear layer with 64 units;
• 1 summation junction with the input to the residual layer;
• 1 ReLU activation.

We remark that consecutive linear layers in the shared stage cannot be unified in a single linear layer because the residual linear layer contains nonlinear activation functions in the output. We also remark that the output of the last layer of the shared stage has size equal to 1 since it needs to be summed up to the previous velocity *v*_*t*−1_ as per Eq. 11.

The reason for using a shared stage is that, in case an input *u*_*t*_ to the system is available, a secondary multi-layer encoder can be used to extract features from *u*_*t*_ (Lee et al., 2020). The features can be concatenated with those extracted from the state *x*_*t*−1_ and then passed to the shared stage. Given that in this work there are no available inputs, we directly feed the state features to the shared stage.

The same structure as above has been used for the networks $NN_{\theta_{2}}$ and $NN_{\theta_{3}}$. Also for these networks, the output size of the last layer equals to 1 as it is the size chosen for the measurement feature in Eq. 16.

The structure of the network $NN_{\theta_{!}}$ is summarized in Figure 2 together with the interconnections with the other networks $NN_{\theta_{2}}$ and $NN_{\theta_{3}}$ throughout the filtering architecture. The inner structure of the linear residual layer is also represented in the bottom left part of the same figure.

#### 4.4.2 Noise Covariance Matrices

Following prior work (Lee et al., 2020), we directly learn the Cholesky decomposition of the noise covariance matrices $L_{Q}\in R^{2\times 2}$ and $L_{R}\in R^{1\times 1}$. In order to take into account this choice, we evaluate the covariance matrix *Q* as $L_{Q}L_{Q}^{T}$ and the covariance matrix *R* as $L_{R}L_{R}^{T}$, according to the definition of the Cholesky decomposition, in the actual implementation of the Kalman prediction and correction steps in Eqs. 9, 10.

### 4.5 Training Protocol

We use backpropagation through time to train our object tracking algorithm end-to-end over subsequences of increasing length *T*_*S*_. Specifically, we perform the training according to the following protocol:

• 5 epochs with *T*_*S*_ = 2 steps;
• 5 epochs with *T*_*S*_ = 4 steps;
• 5 epochs with *T*_*S*_ = 8 steps;
• 5 epochs with *T*_*S*_ = 16 steps;
• 5 epochs with *T*_*S*_ = 32 steps.

### 4.6 Software Implementation

We implemented the software for controlling the iCub robot and collecting the data using the middleware Yet Another Robot Platform (YARP) (Metta et al., 2006). The differentiable EKF has been implemented using the open-source library for creating and training differentiable Bayesian filters in PyTorch (Paszke et al., 2019) from the authors of (Lee et al., 2020). Our software implementation will be made publicly available for free with an Open Source license online^1^.

## 5 Results

In this section we present the results of several experiments aimed at evaluating the performance of the proposed method.

We executed controlled sliding experiments as described in Section 4.2.1 using three objects of different shape and materials in order to collect data for training and testing purposes. We then post-processed the data, as per Section 4.3, and used it to train the proposed differentiable filter for object position and velocity tracking. We evaluated the performance of the algorithm on both the training and testing sequences in terms of the Root Mean Square Error (RMSE) of the estimated state with respect to the ground truth position and velocity. Additionally, we provide the results of several ablation studies aimed at assessing:

• Possible changes in performance when a subset of the x, y and z tactile channels is used instead of the full set;
• The necessity of using the derivative of the tactile measurements $\dot{\tau }$ instead of the plain measurements *τ* as we hypothesized in Section 3.5.
• The generalization capabilities of the algorithm when trained using data of one object in the group and tested on the remaining ones;
• The relevance of the object weight in the training procedure.

Qualitative results on position and velocity tracking performance and considerations on the training and online inference times are also provided.

In a second set of experiments, we tested the possibility to use the output of the learned filter in a practical application. Specifically, we performed the same controlled sliding experiment, as described in Section 4.2.1, while altering the default control commands in order to stop the object sliding after a pre-defined number of centimeters decided by the user. To this end, we compare the positional estimate, provided by the differentiable filter, to a threshold in order to decide when to stop the object sliding by increasing the grasp strength.

Overall, the aim of our experiments is not only to assess the ability of the training procedure to provide reasonable performance on the task of position and velocity tracking during sliding but also the effectiveness of the proposed method in a robotic scenario where the output of the learned filter is used to take decisions on the grasping strength using tactile data solely.

### 5.1 Data Collection

**FIGURE 6:**
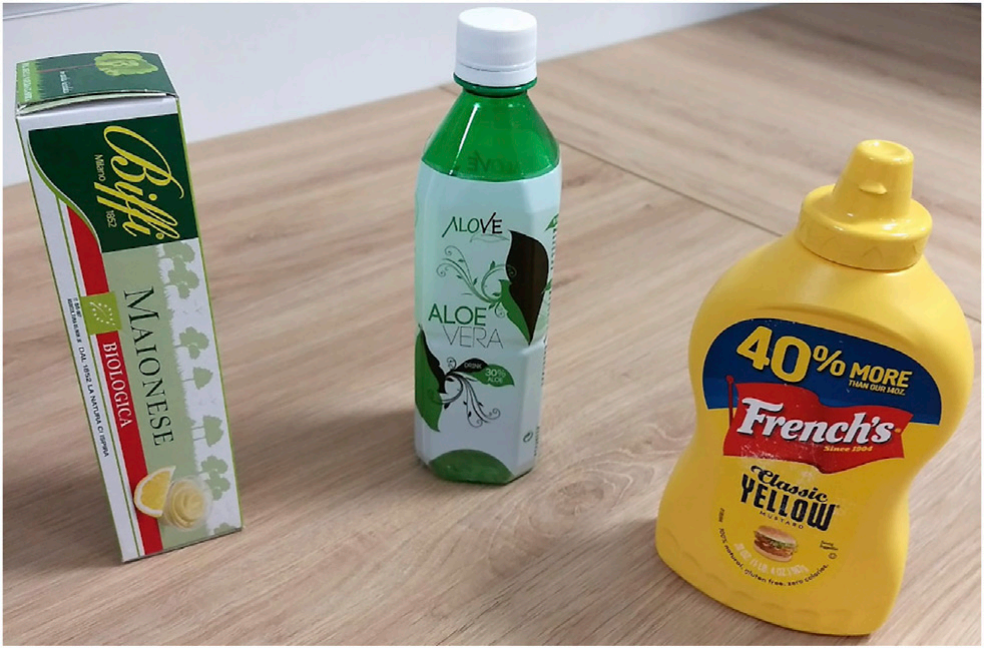
Picture of the objects used in the experiments. From the left, the box-shaped object, the water bottle and the mustard bottle.

The objects that we adopted in our experiments are shown in Figure 6. From the left:

• a box-shaped object made of paper;
• a water bottle made of plastic;
• a mustard bottle made of rigid plastic;

The bottles have been filled such that their weights is approximately 180 g. This weight has been chosen such that it is within that maximum allowed payload for the wrist of the iCub robot hand. Furthermore, this weight allowed us executing the controlled sliding experiments while avoiding excessive maximum normal forces between the fingertips and the object at the moment of stopping the sliding motion. Given that the box-shaped object is made of paper, hence more delicate, we reduced the weight to 100 g in order to reduce the exchanged forces at the moment of stopping the sliding motion and avoid altering its shape. ArUco markers have been applied on all the objects in order to track their position over time as discussed in Section 4.2.3. The collected data will be made publicly available for free online^2^.We executed 100^3^ experiments of controlled sliding for each object with the purpose of collecting training and testing data. Each experiment has been collected at 30 Hz and lasts approximately 1 min. We used half of the sequences for each object as training data and the remaining sequences for testing. The maximum and minimum values *τ*_*g*,*min*_ and *τ*_*g*,*max*_ that we adopted for the three objects are:

• 300 and 600 for the box-shaped object;
• 800 and 1300 for the water bottle;
• 800 and 1500 for the mustard bottle.

**FIGURE 7:**
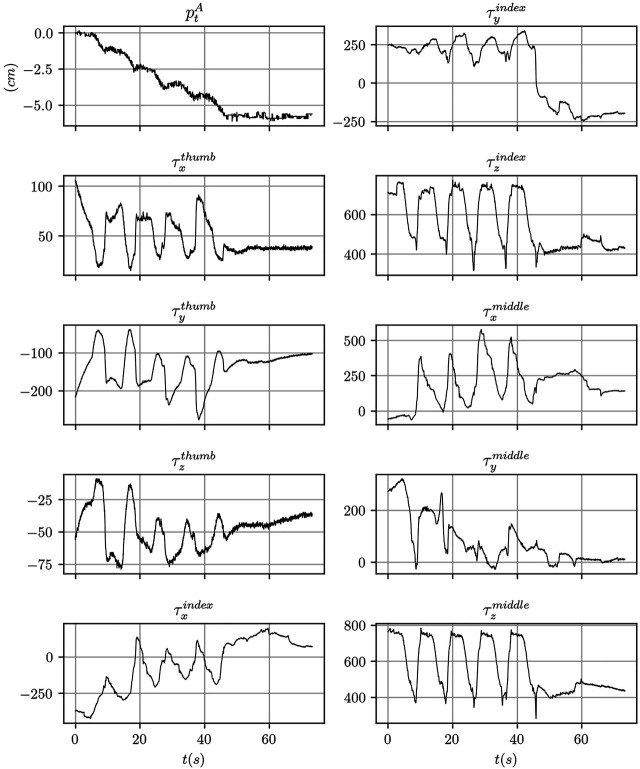
Data traces for one of the data collection experiment performed using the mustard bottle. The tactile signals correspond to the raw sensor reading using arbitrary units.

Sample signals from the ArUco marker detection system and of the tactile sensors output are shown in Figure 7.

### 5.2 Results on Position and Velocity Tracking

#### 5.2.1 Evaluation Metrics

In order to evaluate the performance on pose and velocity tracking we adopted the Root Mean Square Error (RMSE) metric (Bar-Shalom et al., 2002) over the entire trajectory averaged on a set of *N* trials. Given the positional error at time *t* for the *i*-th trial

$$e_{p,i}\left(t\right)=p_{t,i}-p_{t,i}^{gt},$$ (33)

the RMSE is defined as:

$$RMSE\left(e_{p}\right)≔\frac{1}{N}\overset{N}{\underset{i=1}{\sum }}\left(\sqrt{\frac{1}{T}\overset{T}{\underset{t=1}{\sum }}e_{p,i}{\left(t\right)}^{2}}\right)$$ (34)

The error RMSE (*e*_*v*_) on the velocity part is similarly defined.

We also considered the mean of the maximum absolute error over the entire trajectory averaged on a set of N trials as an evaluation metric. For the positional part, it is defined as:

$$max\left(e_{p}\right)≔\frac{1}{N}\overset{N}{\underset{i=1}{\sum }}\underset{t}{\max}|e_{p,i}\left(t\right)|$$ (35)

The maximum error *max* (*e*_*v*_) on the velocity part is similarly defined.

#### 5.2.2 Training and Testing Errors

In Table 1, we report the RMSE and max errors on position and velocity tracking for each object on the training and testing sets. For each object we trained the differentiable filter using data belonging to the training set of that object only. The numerical results show that the training RMSE error in position is below 0.5 cm on average with a maximum average error of 0.845 cm. The performance degrades only slightly on the testing set: the testing RMSE error is below 0.6 cm on average, that we deem as fairly accurate, with a maximum average error of 1.117 cm.

**TABLE 1:** Position and velocity RMS and maximum training and testing errors using the full (*xyz*) set of the tactile channels for all the fingers.

	Training error using *xyz* tactile channels				Testing error using *xyz* tactile channels			
	*e*_*p*_ (cm)		*e*_*v*_ (cm/s)		*e*_*p*_ (cm)		*e*_*v*_ (cm/s)	
Metric	RMSE	max	RMSE	max	RMSE	max	RMSE	max
Bottle	0.200	0.413	0.024	0.086	0.264	0.548	0.028	0.110
Mustard	0.715	1.419	0.048	0.212	0.977	1.882	0.063	0.286
Box	0.422	0.702	0.025	0.109	0.460	0.811	0.034	0.142
Mean	0.446	0.845	0.032	0.136	0.567	1.080	0.042	0.179

The training RMSE error in velocity is below 0.04 cm/s with a maximum average error of 0.136 cm/s. The performance slightly reduces on the testing set with RMSE errors below 0.05 cm/s and a maximum average error of 0.179 cm/s.

We notice that the best performance is achieved with the water bottle object, while for the mustard bottle it degrades more than other objects. We found experimentally that this condition depends on the fact that the sliding experiment has a larger variability in its outcome for this object than others once we fixed the trajectory *τ*_*g*_ of the generalized grasp strength over experiments trials.

#### 5.2.3 Relevance of Tactile Channels

In Table 2 we considered the outcome of several training experiments where we do not feed the entire tactile channel set, i.e. $\left\{\tau_{x}^{f},\tau_{y}^{f},\tau_{z}^{f}\right\}$ for each finger *f*, but only a subset. We recall that, given the choice of the reference frame attached to each tactile sensor (Figure 1), we are expecting that the actual information on the sliding motion is stored in the *x* and *y* channels. On the other hand, the information stored in the *z* channel, while still useful in general, should not be necessary to estimate the sliding motion.

**TABLE 2:** Position and velocity RMS and maximum testing errors using several configurations of the tactile channels (*xyz*, *xy* and *z*).

	Testing error											
	*e*_*p*_ (cm)						*e*_*v*_ (cm/s)					
	RMSE			max			RMSE			max		
Channels	*xyz*	*xy*	*z*	*xyz*	*xy*	*z*	*xyz*	*xy*	*z*	*xyz*	*xy*	*z*
Bottle	**0.264**	0.284	0.674	**0.548**	0.549	1.320	**0.028**	0.030	0.056	**0.110**	0.117	0.152
Mustard	0.977	**0.706**	0.754	1.882	**1.423**	1.613	**0.063**	0.067	0.084	**0.286**	0.301	0.303
Box	**0.460**	0.492	0.442	0.811	0.811	**0.810**	**0.034**	0.038	0.042	**0.142**	0.147	0.148
Mean	0.567	**0.494**	0.623	1.080	**0.928**	1.248	**0.042**	0.045	0.061	**0.179**	0.188	0.201

A bold entry indicates the best result among the available alternatives.

As can be seen from the numerical results on the testing set, the performance achieved using the *xy* configuration is similar to that achieved using the *xyz* configuration but slightly better in terms of positional error. On the other hand, the velocity error is lower for the *xyz* configuration but the difference is less remarkable. The numerical results also show that if we only feed the *z* channel to the differentiable filter, the performance degrades such that for two objects out of three the maximum error in position is more than 1 cm.

**FIGURE 8:**
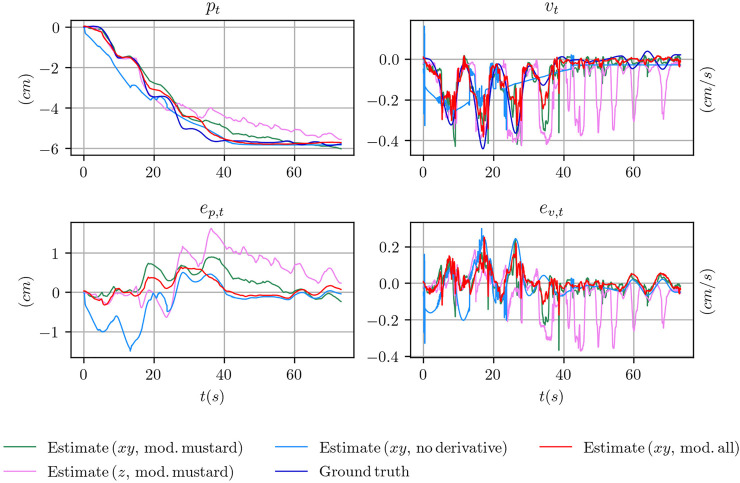
Comparison of the position and velocity trajectories for several configuration of the tactile measurements with the ground truth state for the object mustard bottle.

The fact that the filter is able to produce a reasonable estimate even when using only the *z* channel depends on the fact that the evolution of this channel is constrained by the closed-loop controller, as we explained in Section 4.2.1. When the maximum generalized grasp strength *τ*_*g*,*max*_ is commanded, the object should not slide. Viceversa the object slides, with increasing velocity, when the commanded signal shifts towards *τ*_*g*,*min*_ (Figure 3). However, in practice, it might happen that even when the minimum grasp strength is commanded, the object does not slide or barely slides (e.g. because it touches parts of the fingers or of the hand that prevents the sliding motion). In these cases, using the information stored in the *z* channel only, at training time, would make the training procedure ill-posed. Indeed, the same configuration of the *z* channel would be associated to two totally different outcomes. As an example, when the minimum grasp strength is commanded there will be trials where the associated ground truth velocity is mostly zero and other trials where it is non-zero. This condition might explain why the performance achieved using only the *z* channel is still reasonable but worst than the other configurations. In Figure 8, where we report qualitative results on position and velocity tracking within one experiment from the testing set, it can be seen that using the *z* channel only might actually produce non-zero estimated velocities when the object is actually non sliding, which is undesirable.

Given the above reasoning, the overall outcome of the results reported in Table 2 is that the best channel configuration in terms of positional error is the *xy* configuration. Furthermore, by excluding the *z* channel we reduce the probability of feeding the differentiable filter with ill-posed input-output pairs at training time, which is undesirable.

#### 5.2.4 Necessity of the Derivative of the Tactile Measurements

In Table 3 we report the numerical results of several training experiments where we feed the differentiable filter with the raw tactile measurements *τ* instead of its derivative $\dot{\tau }$ as discussed in Section 3.5. For these experiments we used the final configuration with the *xy* tactile channels as discussed in the previous sections.

**TABLE 3:** Comparison between position and velocity RMS and maximum testing errors when using plain tactile measurements as compared with their time derivative.

	Testing error using *xy* tactile channels							
	*e*_*p*_ (cm)				*e*_*v*_ (cm/s)			
	RMSE		max		RMSE		max	
Measurement	*τ*	$\dot{\tau }$	*τ*	$\dot{\tau }$	*τ*	$\dot{\tau }$	*τ*	$\dot{\tau }$
Bottle	0.381	**0.284**	0.648	**0.549**	0.033	**0.030**	**0.109**	0.117
Mustard	2.698	**0.706**	4.045	**1.423**	0.107	**0.067**	0.401	**0.301**
Box	0.699	**0.492**	1.199	**0.811**	0.049	**0.038**	0.158	**0.147**
Mean	1.259	**0.494**	1.964	**0.928**	0.063	**0.045**	0.223	**0.188**

A bold entry indicates the best result among the available alternatives.

The numerical results demonstrate the necessity to adopt the derivative of the tactile measurements instead of the plain measurement. The RMSE positional error is reduced by ∼60*%* on average and the maximum error by ∼50*%*.

Although the performance degrades considerably, it seems from the numerical results that it is still possible to train the pose and velocity tracking task using the plain tactile measurements. However, as shown in Figure 8, the actual shape of the estimated signal, while it tries to resembles the ground truth, is different in certain key aspects. At the beginning of the experiment, when the object is still not sliding, the slope of the estimated position is wrong and it reaches a non zero position almost instantaneously in a neighbourhood of *t* = 0. This is confirmed by the estimated velocity where, at *t* = 0, an initial spike in the velocity is reported. Furthermore, the shape of the estimated velocity does not follow the actual profile of the ground truth velocity while it tries to follow the mean velocity.

#### 5.2.5 Generalization Capabilities

In Table 4 we report the outcome of several experiments where we tested the possibility of running the learned filter on a given object using the model trained on a different one. For these experiments we used the final configuration with the *xy* tactile channels as discussed in the previous sections.

**TABLE 4:** Position and velocity RMS testing errors using different combinations of training and testing sets. The model name “all” indicates a training set consisting in the union of the training sets of all the objects.

	Testing error using *xy* tactile channels							
	RMSE *e*_*p*_ (cm)				RMSE *e*_*v*_ (cm/s)			
Trained on	Bottle	Mustard	Box	All	Bottle	Mustard	Box	All
Tested on
Bottle	**0.284**	0.600	2.040	0.519	**0.030**	0.080	0.092	0.041
Mustard	0.978	0.706	2.930	**0.664**	0.077	0.067	0.114	**0.066**
Box	0.800	0.821	**0.492**	0.676	0.062	0.067	**0.038**	0.051
Mean	0.687	0.709	1.821	**0.620**	0.056	0.071	0.081	**0.053**

A bold entry indicates the best result among the available alternatives.

Using the model trained on the data of objects bottle and mustard, the average performance on the RMSE positional error is in the order of 0.7 cm. By comparing with the performance in the ideal case, where we test on each object using the correct model (as in Table 2), we can see that the RMSE positional error approximately increased by an amount of 0.2 cm which we consider as a good compromise.

Conversely, if we use the model trained on the box the performance on the other two objects degrades consistently with per object RMSE errors above 2 cm and overall RMSE error above 1.8 cm. This outcome can be explained by the fact that the grasping strength regime for this object (between *τ*_*g*,*min*_ = 300 and *τ*_*g*,*max*_ = 600) is different enough as compared to that of the other two objects. Even if we excluded the *z* channel from the training set, nothing prevents the normal interaction between the fingertip and the object surface to partially project also on the *x* and *y* channels.

Nevertheless, in Table 4 we show that it is possible to train the differentiable filter on all the objects of interest (indicated as *all* in the table) and achieve the best performance. While this configuration requires collection of training data for all the objects of interest, it allows using a single model online without the necessity to know the identify of the object being manipulated by the robot.

In Table 5, we report the results of another experiment aimed at understanding the relevance of the object weight in the training procedure. We considered the scenario in which the object has weight *w*_*target*_ and the training data is available for two neighbouring weights *w*_1_ and *w*_2_, such that *w*_1_ < *w*_*target*_ < *w*_2_. In this experiment, the network is trained using data corresponding to the weights *w*_1_ and *w*_2_ and object position and velocity tracking performance are then assessed using the testing data corresponding to the target weight *w*_*target*_. As baseline, we compare to the case in which the network is trained using the data corresponding to the target weight *w*_*target*_.

**TABLE 5:** Position and velocity RMS testing errors for the box-shaped object with a weight of 125 g when training using data corresponding to the target weight (125 g) or the combination of weights (100 and 150 g).

	*e*_*p*_ (cm)				*e*_*v*_ (cm/s)			
	RMSE		max		RMSE		max	
Channels	*xyz*	*xy*	*xyz*	*xy*	*xyz*	*xy*	*xyz*	*xy*
Trained on
125 g	0.490	0.394	0.929	0.7741.121	0.041	0.046	0.156	0.155
100 and 150 g	0.521	0.615	1.077	0.7741.121	0.071	0.074	0.276	0.368

We executed the aforementioned experiment using the box-shaped object and considered as weights *w*_1_ = 100 g, *w*_2_ = 150 g and *w*_*target*_ = 125 g. 50 experiments were collected for each weight, 10 of which were used for testing. In order to have a fair comparison with the baseline configuration, we fixed the number of experiments used for training. Specifically, when training using the target weight, we used all the 40 experiments designated as training data. When training using the neighbouring weights, we used 20 experiments from the training data corresponding to the weight *w*_1_ and 20 experiments corresponding to other weight *w*_2_. We executed the tracking experiment using both *xy* and *xyz* tactile channels configurations, in order to discuss potential effects of using also the *z* channel when the weight at test time is different from the weight at training time.

As can be seen from Table 5, the tracking performance achieved when using the model trained on the neighbouring weights remains quite acceptable, especially in the case of the *xyz* configuration. In this configuration, the RMSE and maximum positional errors increase by approximately 0.03 and 0.15 cm respectively with respect to the baseline, while the RMSE and maximum velocity errors increase by approximately 0.03 cm/s and 0.12 cm/s respectively. In the case of the *xy* configuration, the drop in performance is more noticeable: the RMSE and maximum positional errors increase by approximately 0.22 and 0.35 cm, while the RMSE and maximum velocity errors increase by approximately 0.03 cm/s and 0.21 cm/s. The results in the same table show that using the *xy* configuration still represents the most indicated solution if training data of the target weight is available (confirming the results discussed in Section 5.2.3). On the other hand, using the *xyz* configuration helps generalizing to weights unseen at training time.

#### 5.2.6 Qualitative Results

In Figure 8 we provide qualitative results on position and velocity tracking for one of the experiment involving the mustard bottle in several configuration of the differentiable filter. Specifically, we compare the ground truth signal with the estimates obtained using the *xy* tactile channels in three cases:

• using the model trained on the data from the mustard experiments;
• using the model trained on all the objects, indicated as *all*;
• using the plain tactile measurements instead of their derivatives.

We also compare with the model trained on the data from the mustard experiments in the case of using the *z* tactile channel only. We report the evolution over time of the position *p*_*t*_ and velocity *v*_*t*_ and their errors *e*_*p*,*t*_ and *e*_*v*,*t*_.

Overall, the configuration that best follows the actual profile of the object position and velocity is given by the model trained on all the data. The model that uses only the *z* tactile channel fails in estimating the correct velocity in the final part of the experiment. Conversely, the model using the plain tactile measurements fails to estimate the correct position in the initial part of the experiment and completely fails to estimate the actual velocity of the object.

#### 5.2.7 Training and Inference Time

Although our software implementation uses the GPU-enabled machine learning framework PyTorch (Paszke et al., 2019), we executed our experiments using CPU computations, instead of a GPU, as we found that both training and inference time were lower. This outcome is expected given that our application does not involve images as input to the network architecture, which typically require the use of GPUs to reduce both training and inference times.

Taking into account the number of experiments whose data is used for training (Section 5.1) and the adopted training protocol (Section 4.5), the training procedure for a single object completes in 22 min and 55 s. The time required for smoothing both the training and testing data, as per the Section 4.3.1, amounts to 52 s while in order to evaluate the derivatives of the tactile measurements for all the experiments, as per the Section 4.3.2, the required time amounts to 5 min and 14 s.

Regarding inference times, the overall filtering pipeline can run at 119 Hz (including the time required to evaluate the derivatives of the tactile measurements), providing real-time state feedback for robot control purposes.

All the presented experiments were executed on an Intel i7-9750H multi-core CPU.

### 5.3 Results on Using the Learned Filter in a Practical Scenario

In this section, we discuss the possibility of using the learned filter in a pratical scenario to take decisions based on the state of the object *x*_*t*_, while it is sliding. We considered the scenario in which the object needs to slide by a given amount of centimeters, indicated as *p*_*target*_, specified by the user. To this end, we adopted the same control architecture for controlled object sliding that has been described in Section 4.2. However, instead of alternating between the maximum grasp strength *τ*_*g*,*max*_ and the minimum grasp strength *τ*_*g*,*min*_ indefinitely until the end of the trajectory, as in Figure 3, we use the tracked position *p*_*t*_ from the filter to decide when to stop the object sliding by applying the maximum strength *τ*_*g*,*max*_. The resulting desired grasp strength *τ*_*g*,*des*_ is selected as follows:

$$\tau_{g,des}=\left\{\begin{array}{cc} alternates\;between\;\tau_{g,min}\;and\;\tau_{g,max}\; & ifp_{t}<p_{target}\\ \tau_{g,max}\; & \text{otherwise} \end{array}\right.$$ (36)

#### 5.3.1 Evaluation Metrics

In order to evaluate the performance of the experiment we use the following metrics. We define the *decision error* *e*_*decision*_ as the absolute error between the filtered position *p*_*t*_ and the ground truth $p_{t}^{gt}$ at the instant of time in which *p*_*t*_ ≈ *p*_*target*_, averaged on N trials of the experiment. The decision error accounts for the error committed when the maximum grasp strength is applied and the object stops moving, and depends on the positional error of the filter.

Next, we define the *target error* *e*_*target*_ as the absolute error between the real position of the object $p_{t}^{gt}$ and the target position *p*_*target*_ 10 s after the decision to stop the sliding, averaged on N trials. The target error measures the effectiveness of the overall strategy in stopping the motion of the object when the target position is reached.

Finally, we define the *long-term error* *e*_*long*−*term*_ as the absolute error between the filtered position *p*_*t*_ and the ground truth $p_{t}^{gt}$ 10 s after the decision. This error evaluates the ability of the filter to remain stable after the maximum grasp strength has been applied. This ability is not necessarily granted as the desired grasp strength, modified as in Eq. 36, has not been adopted at training time.

#### 5.3.2 Discussion of the Results

We executed 20 experiments for each object with different targets *p*_*t*_, namely −1.5 cm for the box and −2.5 for the two bottles. The experiments have been executed using the tactile *xy* channels. In the case of the box, we also repeated the experiment using the full *xyz* configuration in order to discuss potential effects of this configuration on the tracking performance in presence of grasping strength profiles not seen at training time.

**FIGURE 9:**
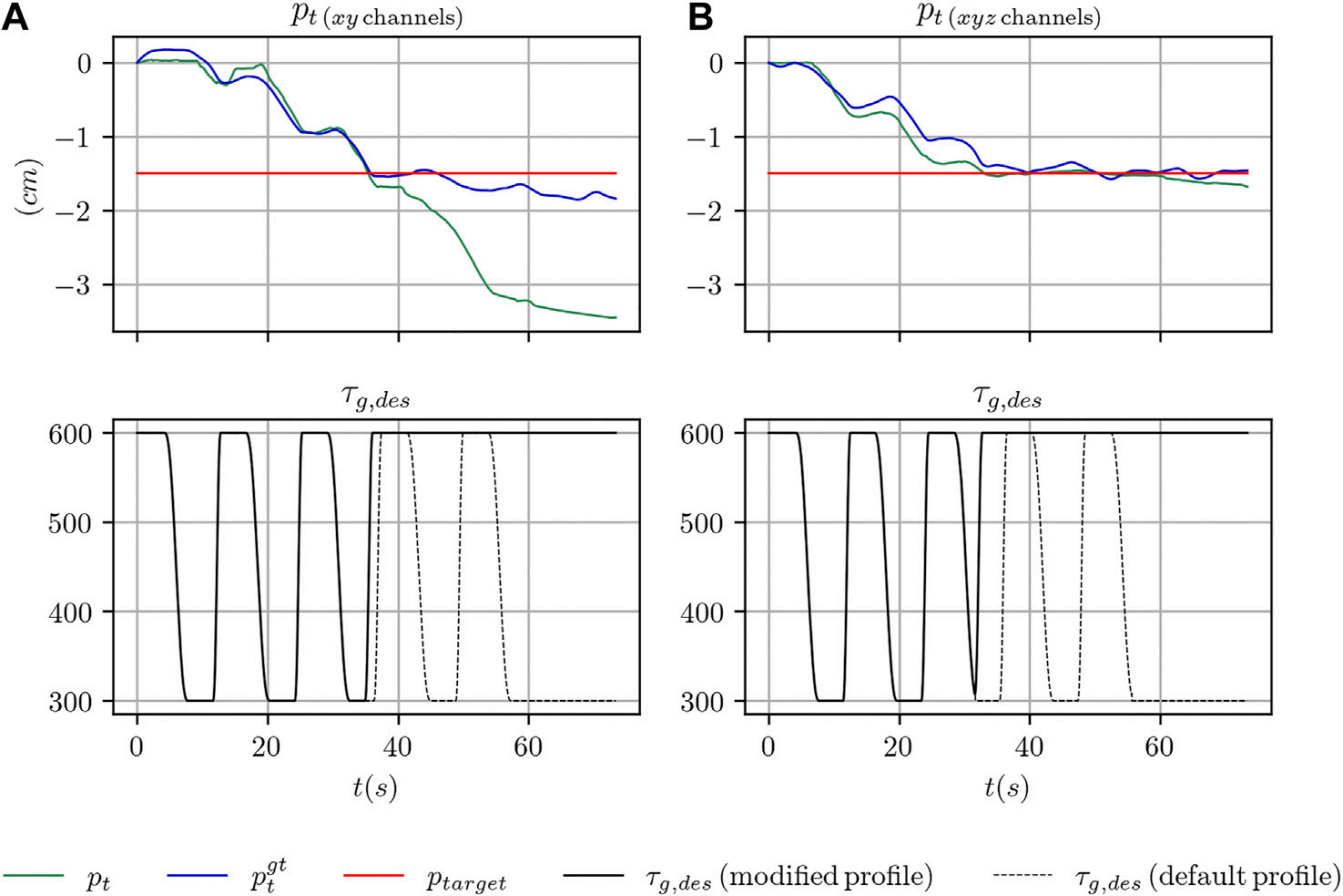
Sample trajectories from one of the experiments on using the learned filter in a practical scenario with the box-shaped object. In **(A)**, the results are shown when using the *xy* tactile channels, in **(B)** when using the *xyz* tactile channels.

In Table 6 we report the results of the experiment in terms of the errors *e*_*decision*_, *e*_*target*_ and *e*_*long*−*term*_. As can be seen, the decision and target errors are in the order of fraction of centimeters and not higher than 0.66 cm. These results are in accordance with our findings on the position tracking performance and suggest the effectiveness of the proposed solution if the requested precision is in the order of approximately half a centimeter or higher. Conversely, the long term errors are slightly higher, especially for the mustard and the box-shaped objects. This fact is also confirmed by the qualitative results that we report in Figure 9.

**TABLE 6:** Decision, target and long-term errors for several objects with different weights, several targets and configurations of the tactile channels.

Object	Bottle	Mustard	Box	
Target	−2.5 cm	−2.5 cm	−1.5 cm	
Weight	180 g	180 g	100 g	
Tactile channels	*xy*	*xy*	*xy*	*xyz*
*e*_*decision*_ (cm)	0.459	0.656	0.330	0.216
*e*_*target*_ (cm)	0.386	0.547	0.274	0.207
*e*_*long*−*term*_ (cm)	0.418	1.101	0.748	0.357

Box	
−1.5 cm	
125 g	125 g (Trained on 100 and 150 g)
*xyz*	
0.160	0.312
0.223	0.409
0.181	0.281

On the left side of the figure we report the outcome of one experiment with the box-shaped object in the case of using the *xy* channels of the tactile sensors. After the sliding motion of the object is stopped and the desired strength changed to the maximum value *τ*_*g*,*max*_, the filter starts diverging over time.

On the right side of the same figure, we report the outcome of another experiment with the box-shaped object when all the tactile channels of the sensors are adopted. As can be seen, the target position is similarly reached and the output of the filter remains stable even many seconds after the motion of the object has been stopped. This comparison suggests that using the *z* channel at training time, while not crucial for the position tracking performance, as discussed in the previous section, might help generalizing to configurations of the grasp strength profile not seen at training time. Our reasoning is also confirmed by the numerical results in Table 5, where, in the case of the box-shaped object, the long term error reduces by approximately 50*%*.

We also executed several experiments with the box-shaped object when considering different weights as done in Section 5.2.5. We used the *xyz* tactile configuration that helps generalizing to weights and grasp strength profiles unseen at training time, as discussed in the previous sections. Similarly to Section 5.2.5, we trained the network using two neighbouring weights, namely 100 and 150 g, and executed the experiment with a target weight of 125 g. As baseline, we executed the experiment using a model trained on the target weight. We executed 20 experiments for each configuration and we report the averaged metrics in Table 6. As can be seen, even when using a model trained on weights different from the target one, the performance, while degrading with respect to the baseline, remains quite reasonable with decision, target and long-term errors of approximately 0.3, 0.4 and 0.3 cm respectively.

## 6 Assumptions and Limitations

The present work would not have been possible without relying on several assumptions and approximations that we discuss in this section in order to identify aspects to be further investigated in future research.

### 6.1 Handling of Object Rotations

In this work, we intentionally focus on the problem of tracking pure translational sliding motions instead of general slippage motions involving rotations of the object.

Similarly to other works from the slip detection literature (Veiga et al., 2018), our data collection procedure (Section 4.2) is not designed to capture object rotations. While ground-truth data for object translational motions can be easily collected even by using a simple marker and a limited amount of data post-processing, rotations during slippage might be of limited and subtle magnitude, hence more difficult to collect due to the noise in the marker pose detection process. Furthermore, in-hand object rotations heavily depend on the position, eventually discontinuous, of the contact points with respect to the object center of mass and on the inertial properties of the object. However, a precise control of the contact points, useful to achieve a repeatable data collection procedure, is out of scope of the present work. Nonetheless, we deem important to study how to account for object rotations in the proposed architecture and we plan to investigate this in future research.

### 6.2 Handling of External Disturbances

In the present work, we tackle the problem of object sliding tracking in the scenario of in-hand object manipulation in which gravity is the only external force applied to the object. We remark that this scenario is not unrealistic and that it is adopted by other works, e.g. in the literature on tactile-based object manipulation and perception (Meier et al., 2016; Bimbo et al., 2015; Liang et al., 2020; Suresh et al., 2020). We deem it is important to address this scenario first before considering the more general case in which other forces and external disturbances act on the object.

### 6.3 Characteristics of the Considered Objects

In this work, we considered two prism-shaped objects (i.e. the box-shaped object and the water bottle made of plastic) and one object with non-convex shape, i.e. the mustard bottle. The former are characterized by a constant cross section such that the fingers can slide freely on the object surface while avoiding to be blocked by irregular changes in the object shape. While the mustard bottle presents some irregularity in the shape of the lateral edges, we limited our experiments to the case in which the fingers slide along the wider surface of the bottle. Although we believe that tracking the sliding motion along irregular surfaces would be useful, we recognize that this problem is connected more with the ability of the fingers controller to follow an irregularly-shaped surface rather than the ability of the filtering architecture to process the signal produced by the sensors in that scenario.

Regarding the size, and especially the length, of the objects, in this work we considered objects having at least one direction with a non-negligible length (see Figures 1, 4) as compared to that of the palm of the hand of the adopted robotic platform, i.e. the iCub humanoid robot. The reason behind this choice is mostly practical, as we are interested in the problem of estimating the sliding motion of the object, hence it is required to have objects that could actually slide between the fingers of the hand for a certain number of centimeters. We remark that this assumption does not prevent the possibility to use the proposed pipeline with objects that are smaller than those adopted in our experiments, as long as the desired tracking precision stays within the average position error achieved by our pipeline, i.e. 0.6 cm.

### 6.4 Application to Closed-Loop Control

In the experimental section of this work, we provide the results of a simple practical application where the output of the proposed pipeline is used to stop the sliding motion of the object after a certain number of centimeters provided by the user. We use a simple thresholding mechanism to achieve this behavior. A natural question arising from our experiments is whether it is possible to control the motion of the fingers continuously, using the feedback from the learned filter, in order to control the sliding velocity of the object and track a desired reference velocity. Furthermore, we deem important to understand whether the learned differentiable motion model in Eq. 11 can be utilized in the synthesis of the aforementioned control system using model-based control techniques. We plan to investigate these aspects in future work.

## 7 Conclusion

In this paper we proposed an approach for tracking the 1-D position and the velocity of an object undergoing translational sliding motion between the fingers of an anthropomorphic hand equipped with tactile sensors. We showed how to collect clean labelled data directly on a real humanoid robot and train a differentiable Extended Kalman filter end-to-end for the task of position and velocity tracking.

Experiments using a real anthropomorphic hand equipped with tactile sensors, and integrated on the iCub humanoid robot platform, show that our approach allows achieving position tracking errors in the order of 0.6 cm on average, and that the proposed method can be used effectively to control the sliding of the object using tactile feedback alone.

As future work, we propose to extend the presented algorithm in order to take into account the possible 3D rotation of the object during the sliding motion. Furthermore, we propose to the utilize the learned motion and measurement models to synthesize a controller for precise object velocity control during the sliding motion.

## Data Availability

The datasets presented in this study can be found in online repositories. The names of the repository/repositories can be found below: https://github.com/robotology/dekf-tactile-filtering.

## Footnotes

1
https://github.com/robotology/dekf-tactile-filtering

2
https://github.com/robotology/dekf-tactile-filtering

3 The number of experiments for the object “mustard bottle” amounts to 75.
